# Dental Care in Times of the COVID-19 Pandemic: A Review

**DOI:** 10.3390/medsci9010013

**Published:** 2021-02-19

**Authors:** Erfan Shamsoddin, Lisa M. DeTora, Marcos Roberto Tovani-Palone, Barbara E. Bierer

**Affiliations:** 1 Cochrane Iran Associate Centre, National Institute for Medical Research Development, Tehran 02166, Iran; erfanshamsoddin@gmail.com; 2 Department of Writing Studies and Rhetoric, Hofstra University, Hempstead, NY 11549, USA; lisa.m.detora@hofstra.edu; 3 Ribeirão Preto Medical School, University of São Paulo, Ribeirão Preto 14049-900, Brazil; 4 Brigham and Women’s Hospital, Harvard Medical School, Boston, MA 02115, USA; bbierer@bwh.harvard.edu

**Keywords:** public health dentistry, COVID-19, saliva, telehealth, triage

## Abstract

Given the dynamic relationship between oral and general health, dental care must not be neglected even during a public health emergency. Nevertheless, the fear of contracting the infection appears to have caused instances of dental treatment avoidance. In these times of uncertainty, regulatory and public health organizations have made numerous and sometimes controversial recommendations to practitioners and to the public about how to secure their oral health care needs. Dentists, as advocates of oral health, should actively maintain their practices while considering local epidemiological reports and recommendations regarding prevention of SARS-CoV-2 infection. Providing appropriate safety measures, accurate triage and prioritization of patients, notice to susceptible communities, remote health care delivery when appropriate, and epidemiological reports of COVID-19 (whenever possible) are all critical considerations for dental practitioners.

## 1. Highlights

- Effective triage and prioritization are essential for dental practitioners during health emergencies. Local epidemiological recommendation should be followed by all dentists and their practices.
- Dental health care is not limited to in-office clinical treatment. Patients should be supported by remote dental care (teledentistry, online visits and prescriptions, etc.) whenever possible.
- Dentists should be aware of the most up-to-date or current COVID-19-related findings with regard to diagnostic and prognostic features of the oral cavity and its components (e.g., saliva, tongue, etc.).

## 2. Background

Assessing information-seeking behavior during the Coronavirus disease 2019 (COVID-19) pandemic substantiates the idea that public anxiety and fear of severe acute respiratory syndrome coronavirus 2 (SARS-CoV2) infection led to avoidance of dental treatment [1,2,3]. This observation is further supported by clinical narratives among dentists describing an epidemic of cracked teeth, associated with COVID-19-related stress and insomnia among the general population, causing more chewing pain or discomfort [4]. An additional barrier to seeking timely dental care is the perception of dentistry as stressful [5]. As a result, routine dental care is often delayed, and patients may address their emergency needs using inadvisable alternatives. However, clinicians still can provide dental care safely if they follow infection prevention guidelines and applicable clinical guidelines for the delivery of routine and emergency dental care to both uninfected individuals and COVID-19 patients while reducing the chance of infection in dental patients, dental practitioners, and their staff. With the use of virtual technologies, teledentistry, and the provision of online care, remote help and appropriate advice can be provided. Virtual visits help to ensure that only those patients who need timely procedures are referred to a clinical site for care. Universal and other precautions permit necessary office and emergency visits. Effective triage and use of dental visits may also assist in developing public health reports with regard to COVID-19 exposure history. Aiming to improve the management dental patients (inside and outside of dental offices), we review the importance, criteria, and guidelines for dental treatment during the current pandemic.

## 3. Importance of Oral Health

The COVID-19 pandemic delayed access to necessary medical treatment (e.g., chemotherapy) and routine care, such as vaccination [6,7]. Oral health, a good indicator of overall health, has also been affected by this public health emergency. Poor oral health can portend other systemic diseases, including atherosclerosis, pulmonary disease, diabetes, pregnancy, low birth weight, osteoporosis, and kidney disease [8,9]. The teeth, periodontium, and biofilm can serve as reservoirs for pathogens and may facilitate pathogen reproduction in the lungs by aspiration even in healthy individuals [10]. The risk of infection is of even greater concern in critically ill patients with limited biological defenses, those who are therefore more susceptible to ventilator-associated pneumonia (VAP) [8]. Recent evidence has argued for the benefit of oral health care against VAP, especially in black patients [11].

Timely dental care and appropriate follow up can prevent more serious disease not only in the oral cavity but also systemically [12,13,14]. Pulpitis, for instance, may rapidly become irreversible, causing a constant, dull, and throbbing pain [15]. Acute periapical dental abscesses may progress to septicemia or respiratory obstruction and an increased risk of mortality [16]. Periodontal and endodontic problems have been shown to alter the course of bacterial pneumonia, cardiovascular diseases, and diabetes mellitus. Moreover, if these oral problems occur during pregnancy, they may be associated with neonates of low birthweight [17]. Whether and how poor oral health contributes to health disparities observed in the incidence and prevalence of COVID-19 has yet to be addressed [18]. Considering the essential place of oral health to overall health, as well as the persistence of the SARS-CoV-2 virus (and the emergence of new variants), it is imperative to take further heed of how dental care will continue to be affected by the COVID-19 pandemic [19]. Significant changes in the routine dental care provision and prioritizing dentists to get the COVID-19 vaccine in the first phase of distribution in 40 states of the US are all harbingers of this long-lasting influence and the rapid adaptations made by the profession [20].

## 4. Prioritizing Dental Care

It may seem paradoxical to provide dental treatment while attempting to limit the spread of an airborne infectious diseases. The World Health Organization (WHO), the Centers for Disease Control and Prevention (CDC) guidance on oral health services, and the Cochrane community recommended delaying routine dental care [21,22,23]. The American Dental Association (ADA), however, advised against the adoption of these recommendations [24]. This disagreement highlights the differences between a broadly public-health oriented perspective and the importance of specialist knowledge during a public health emergency (PHE). A balanced perspective between these positions is required to effectively address the needs of public health. Several published dental health care protocols agree that providing emergency and urgent dental care for issues such as swelling, pain, bleeding, dental trauma, and invasive infections should be prioritized [2,19,25,26,27]. Virtual technology or telephone screening is advised before scheduling in-person visits or admitting patients to the health care facility [19]. Comprehensive exposure history to screen asymptomatic but infected people may be beneficial for public health reporting as well [19].

Primary triage should provide advice, analgesia, and antibiotics (where appropriate) [24,26]. Most odontogenic pain can be managed effectively using paracetamol (acetaminophen) or ibuprofen [28,29]. Patients with unresolved symptoms after 48–72 h of self-management may then be guided to facilities equipped with adequate measures to limit or halt viral spread. Mobile dentistry services may be warranted for more susceptible patients and geriatric populations [19].

**Figure 1:**
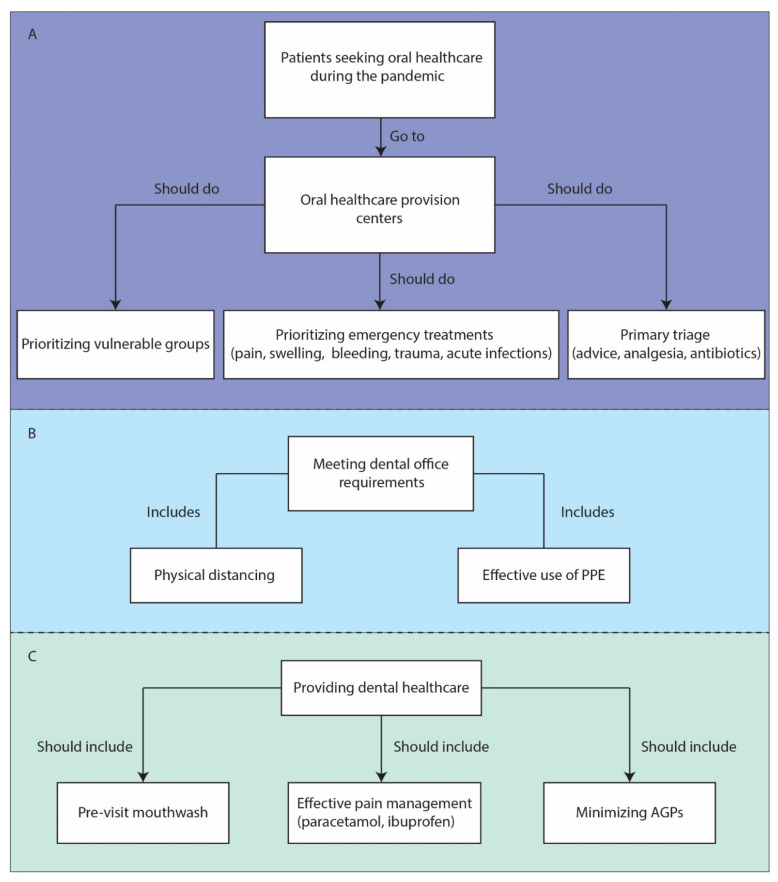
Recommendations for dental practice during COVID-19 pandemic. (**A**) Essential prioritization and triage. (**B**) Essential requirements of active dental offices. (**C**) Essential steps to notice in healthcare delivery. COVID-19: Coronavirus disease 2019; PPE: Personal protective equipment; AGPs: Aerosol generating procedures.

When providing urgently needed dental care, appropriate physical distancing protocols and personal protective equipment (PPE) should be used [19,30]. Minimizing aerosol generating procedures (AGPs), continuous use of face masks, hand and respiratory hygiene, scheduling appointments to promote social distancing, using sterile instruments and devices, and disinfecting environmental surfaces regularly are also recommended [19,26]. Prescribing a pre-visit mouthwash, such as chlorhexidine gluconate (CHX), cetylpyridinium chloride (CPC), povidone-iodine (PVP-I), and hydrogen peroxide (H_2_O_2_), has also been recommended in the literature [31]. Additionally, to help maintain physical distancing, patients should not be accompanied unless medically necessary, and then the companion should be subject to similar screening and protective measures [19]. These recommendations are schematically summarized in Figure 1.

Reorganizing dental offices may be beneficial for infection control as well [23]. Dentists should avoid performing AGPs whenever possible, but high-volume suction devices, protective face masks (N95 or higher protection) and face shields, body suits and eye protection, and shortening procedure duration may mitigate risk for necessary AGPs [19,23,26,32]. Despite its utter importance in providing care, PPE may be more difficult to acquire in resource-poor areas, a significant concern for all dentists [33].

Reopening dental practices should be an adaptive process subject to local epidemiological reports. Protecting the dental health care provision team is also important. Consequently, implementing COVID-19 screening procedures for dental staff prior to arrival at work and flexible, non-punitive sick leave policies are crucial [19]. Furthermore, the exposure risk of patients before and two days after receiving dental treatment should be assessed, as should that of dental staff [19]. Active communication and forming liaisons with other medical practitioners are highly recommended [25]. Even after the arrival and availability of effective vaccines, it will take time to return to a routine state of clinical practice regarding the chances of infection in dental offices; hence universal infection control protocols should be followed continuously until the local health authority determines that it no longer necessary to do so.

## 5. Identifying Susceptible Groups

When addressing oral health needs, susceptible or vulnerable individuals should be prioritized and health care—and access to health care—provided equitably. During a PHE, chronic disease patients, cancer patients, pregnant women, underrepresented minority and marginalized populations, people who are educationally or economically disadvantaged, nursing home residents (geriatric, disabled, or debilitated patients), and incarcerated individuals are all considered to be more susceptible and to need equitable access to oral health care [34,35,36,37,38,39,40]. Further, in these settings, the general health condition of these individuals is often poorer than that of the general population [41]; hence the salience of supporting these patients remotely and treating urgent dental problems. Protecting quality of life necessitates timely dental interventions for vulnerable groups; serious outcomes of treatment delay in susceptible patients are important determinants for active and timely intervention. For instance, adverse pregnancy and birth outcomes, early childhood caries, and chronic diseases are associated with poor maternal oral health conditions [37]. Given that the overall trend in dentistry has been moving towards preventive approaches, providing appropriate dental care in pediatric patients is of utmost importance [42]. Dental management of organ transplant patients before the major operations is another critical need: significant adverse effects, especially infection risk, can result from postponing dental care [43]. Online dental visits and providing motivation for practicing oral hygiene are in line with building an appreciation of the importance of prevention in children. Moreover, anxiety and stress disorders in susceptible individuals could lead to more detrimental effects when dental related complaints rise (e.g., pain, mouth ulcers, bruxism, xerostomia, etc.) [44]. Hence, these groups warrant priority when addressing dental services provided remotely.

## 6. Managing Expectations and Concerns of the Patient and Public

Pain is the most common complaint of patients seeking dental care [45]. Identifying the cause of pain and explaining possible diagnoses may help reduce anxiety [46]. Enhancing clinician–patient communication can also assist in community engagement and education [47]. Whilst some limitations exist with regard to accessing online care and information, wide use of smart phones, and the intrinsic ability to share photographic information when appropriate, has rendered communication easier. Providing up-to-date educational content and resources also increases public understanding. The use of informative flyers and dental guides could inform the patients about etiologies of common symptoms. In addition, preparing COVID-19 related resources may be beneficial for both health care workers and patients. Pre-visit online interviews with patients could reduce anxiety by providing information about any changes they might face during their office visit and treatment [19]. Addressing anxiety concerning infection control is necessary as well. This fear and anxiety might even continue after the introduction of effective vaccines for COVID-19 and needs to be mitigated for better prognoses as patients’ cooperation with the dentist’s instructions plays an important role in achieving better outcomes, especially for periodontal management [48].

## 7. Assisting in Epidemiology of the Pandemic

Saliva has been identified as a valuable diagnostic option for early COVID-19 case detection [49,50]. Oral swabs from COVID-19 patients showed higher positive rates (by RNA content) in early stages of the illness than a number of other test methods. SARS-CoV-2 is capable of invading salivary gland cells via the expression of angiotensin converting enzyme-2 (ACE2) receptors to which the virus binds [51,52]. Additionally, the existence of 2019-nCoV nucleic acid in saliva accessed from the salivary gland has been associated with the severity of COVID-19 [53]. These preliminary findings suggest that saliva may have a role as a diagnostic tool; whether and how diagnostics based on saliva could enhance surveillance is a worthwhile direction for clinical research.

## 8. Summary

Dental practitioners are the advocates of oral health and are in a position to motivate the public to seek both preventative, routine, and emergency oral healthcare during COVID-19 and other PHEs, relying upon safe and available platforms, universal precautions, and timely epidemiological information. Application of teledentistry, attention to infection control measures, and the development of isolated emergency dental care centers are pragmatic and effective ways to maintain and enhance oral health among the public. The post-vaccine era will not likely portend a return to pre-2020 practices; dentists need to continue triage, surveillance, and reporting to halt the viral spread and help identify and track the emergence of new variants. Further research on the diagnostic and prognostic role of saliva in COVID-19 research is recommended as well.

## Data Availability

Not applicable.
